# Comparative Detection of *IDH1* R132H and *TERT* Promoter Mutations in Plasma and Cerebrospinal Fluid of Patients with Glioma Using Targeted Droplet Digital PCR

**DOI:** 10.3390/diagnostics16203333

**Published:** 2026-10-14

**Authors:** Mark Jain, Tagir Rahmatullin, Ivan Alexeev, Ariadna Gomershtat, Margarita Gundobina, Larisa Samokhodskaya, Andrey Zuev

**Affiliations:** 1 University Clinic, Lomonosov Moscow State University, 119992 Moscow, Russia; tagir.rakhmatullin@internet.ru (T.R.); slm61@mail.ru (L.S.); 2 National Medical and Surgical Center Named After N.I. Pirogov, 105203 Moscow, Russia; alexeev.im@yandex.ru (I.A.); mosbrain@gmail.com (A.Z.); 3 Faculty of Fundamental Medicine, Lomonosov Moscow State University, 119992 Moscow, Russia; ariadna.gomershtat@mail.ru (A.G.); gundobinamargarita@gmail.com (M.G.)

**Keywords:** cell-free DNA, liquid biopsy, cerebrospinal fluid, plasma, circulating DNA, *TERT* promoter, *IDH1*, mutations, digital droplet polymerase chain reaction, glioma

## Abstract

**Background/Objectives**: Molecular profiling is essential for glioma classification, but in some cases tissue sampling may be limited, whereas reported detectability of cell-free tumor DNA (cftDNA) in plasma and cerebrospinal fluid (CSF) varies considerably. The aim of this study was to compare the detection rates and concentrations of cftDNA carrying *IDH1* R132H and *TERT* promoter C228T/C250T mutations in paired plasma and CSF samples, as well as to examine associated clinical characteristics. **Methods**: 66 patients with WHO grade 1–4 gliomas were enrolled before surgery. Preoperative plasma and lumbar CSF were collected before tissue-damaging procedures. Cell-free DNA (cfDNA) was isolated from 5 mL of plasma and 2.5–5.0 mL of CSF and analyzed using mutation-specific droplet digital PCR (ddPCR) assays. **Results**: cfDNA was available from 65 CSF and 63 plasma samples. Total cfDNA concentration was lower in CSF than in plasma (medians: 434.1 vs. 1240.3 copies/mL; *p* < 0.001). Nevertheless, cftDNA carrying at least one of the assayed mutations was detected in 14/65 CSF samples (21.5%) and in 0/63 plasma samples (0.0%) (*p* < 0.001). *IDH1* R132H was detected in 4.6% of CSF samples, and *TERT* promoter mutations were detected in 16.9%. Every CSF mutation with available paired tissue data was also identified in the corresponding tumor. Glioma grade 4 and contrast-enhancing tumors were associated with higher CSF cfDNA, cftDNA carrying at least one of the assayed mutations, and mutant allele fraction values (*p* < 0.05), whereas tumor size and localization were not significantly associated with these parameters (*p* > 0.05). **Conclusions**: With the presented targeted ddPCR workflow, lumbar CSF was substantially more informative than plasma for detecting the selected glioma-associated mutations. CSF cftDNA analysis may complement tissue profiling, although broader panels and larger prospective studies are needed to establish its clinical sensitivity.

## 1. Introduction

Primary brain tumors comprise a heterogeneous group of neoplasms that, although relatively uncommon compared with many extracranial malignancies, are associated with substantial morbidity (7.0 per 100,000 population) and mortality (4.4 per 100,000 population) [1]. Gliomas represent a predominant group of malignant primary brain tumors in adults and include genetically and clinically distinct entities ranging from relatively indolent oligodendrogliomas to highly aggressive glioblastoma [2]. The clinical burden of gliomas is further increased by their effects on neurological function, cognition, and quality of life, as well as by the limited effectiveness of currently available treatments for advanced disease [1].

Over the past decade, molecular genetic profiling has become essential to the diagnosis and clinical management of diffuse gliomas. The fifth edition of the World Health Organization Classification of Tumors of the Central Nervous System introduced an integrated diagnostic approach combining histological and molecular characteristics [3]. Adult-type diffuse gliomas are now principally divided into astrocytoma, IDH-mutant; oligodendroglioma, IDH-mutant and 1p/19q-codeleted; and glioblastoma, IDH-wildtype [3,4]. Point mutations in isocitrate dehydrogenase genes, particularly the canonical *IDH1* R132H substitution, constitute early oncogenic events and define biologically distinct glioma groups. Mutant IDH enzymes acquire neomorphic activity, resulting in the production of D-2-hydroxyglutarate and extensive metabolic and epigenetic reprogramming. Therefore, IDH status provides important diagnostic and prognostic information and increasingly influences therapeutic decision-making [5]. Substitutions in the promoter of the telomerase reverse transcriptase (*pTERT*) gene are another major molecular feature of diffuse gliomas in adults. The two recurrent hotspot mutations, *pTERT* C228T and C250T (at −124 bp, chr. 5: 1,295,228, G/A; and −146 bp, chr. 5: 1,295,250, G/A, assembly GRCh37, respectively) generate new transcription-factor binding sites and increase *TERT* expression and, thus, telomerase activity [6]. *pTERT* mutations are particularly frequent in IDH-wildtype glioblastomas and *IDH*-mutant, 1p/19q-codeleted oligodendrogliomas, although their diagnostic and prognostic value depends on the accompanying molecular alterations [7].

Currently, molecular characterization of gliomas is mostly performed using surgically obtained tumor tissue. However, tissue-based genetic profiling has several limitations. Resection or stereotactic biopsy may be difficult or contraindicated, because of tumor localization in deep or functionally eloquent brain structures, an unfavorable risk-to-benefit ratio, or the poor general condition of the patient. Even when a tissue specimen is available, its quantity and quality may be insufficient for comprehensive molecular testing [4,8]. Furthermore, gliomas demonstrate considerable spatial and temporal heterogeneity. A small biopsy may not represent all genetically distinct tumor regions, while molecular evolution under therapeutic pressure may cause the recurrent tumor to differ substantially from the primary lesion [9,10]. Whereas repeated neurosurgical biopsies are generally impractical, limiting the ability of tissue analysis to monitor clonal evolution and emerging treatment resistance over time.

Liquid biopsy has emerged as a potential complementary approach for minimally invasive tumor genotyping. The analysis of tumor-derived components released into biological fluids, particularly cell-free tumor DNA (cftDNA), may provide information about the molecular composition of a tumor without requiring direct tissue sampling [11]. In patients with gliomas, both plasma and cerebrospinal fluid (CSF) have been investigated as sources of cftDNA [12]. Plasma collection is minimally invasive and readily repeatable, making it attractive for longitudinal monitoring. However, the release of glioma-derived DNA into the systemic circulation may be restricted by the blood–brain barrier, limited tumor vascularity, rapid clearance, and dilution by non-tumor cell-free DNA (cfDNA) [13,14]. CSF is anatomically closer to brain tumors and may therefore contain higher proportions of cftDNA, particularly when the lesion contacts the subarachnoid space. Several studies have demonstrated that CSF cftDNA can reproduce tumor-specific genomic alterations and, in some cases, reflect spatial heterogeneity and molecular evolution more comprehensively than a single tissue specimen [15,16,17]. Nevertheless, CSF collection is more invasive than venipuncture and may not be clinically appropriate for every patient.

Published estimates of cftDNA detectability in glioma patients vary considerably. Most sequencing-based studies have reported substantially higher detection rates in CSF than in plasma, supporting the biological advantage of a fluid that is in direct contact with the central nervous system. For example, tumor-derived alterations have been detected in CSF in 50–100% of patients in several glioma cohorts, with higher detection probability associated with tumor burden, progression, proximity to the subarachnoid space, and adverse clinical characteristics [16,18,19]. Reported detection rates of cftDNA in plasma varies significantly as well. For example, Fontanilles et al. detected plasma cftDNA in only 2 of 52 patients with newly diagnosed glioblastoma, both of whom had gliosarcoma [20]. Other researchers have reported substantially higher plasma detection rates for *IDH1* and *pTERT* hotspot mutations [21]. A recent meta-analysis estimated that pooled cftDNA detection rates in CSF are 5.1 times higher than in plasma, but also noted considerable inter- and intra-study heterogeneity which complicated the comparison [17]. These discrepancies may be related to differences in tumor grade, molecular subtype, disease stage, tumor volume and localization, contact with CSF spaces, blood–brain barrier disruption, treatment status, sample volume, processing procedures, analytical sensitivity, and criteria used to define a positive result. Consequently, the relative performance of plasma and CSF remains incompletely established, particularly when both fluids are analyzed within the same clinically characterized cohort using an identical analytical method.

Given these inconsistencies, the present study aimed to compare the detection rates and concentrations of cftDNA in plasma and CSF samples from glioma patients. Additionally, we sought to identify factors potentially associated with qualitative and quantitative characteristics of cftDNA.

## 2. Materials and Methods

### 2.1. General Information

The protocol of this study was critically reviewed and approved by the Ethics Committee of the hospital (protocol No. 3, dated 16 March 2022). The study was conducted in strict accordance with the Declaration of Helsinki and all subsequent amendments. Patient enrollment was performed from May 2022 to April 2024 (*n* = 66).

Inclusion criteria:

- Diagnosis of glioma (WHO grade 1–4);
- Planned total or partial surgical resection of the tumor.

Exclusion criteria:

- History of any other oncological disease;
- Diagnosis of any other oncological disease after study enrollment;
- Neoadjuvant chemotherapy.

Glucocorticoid therapy was not routinely initiated during the preoperative period as part of the study protocol and was not considered an exclusion criterion. However, some patients received glucocorticoids before surgery for clinical indications, particularly for the management of clinically significant peritumoral edema. Intraoperatively, patients received 8 mg of dexamethasone, which was administered only after collection of blood and CSF samples for the present study. Morphological verification of the diagnosis was performed by histopathological evaluation of resected tumor tissue. Visualization and assessment of tumor spatial characteristics were performed using computed tomography, magnetic resonance imaging, and positron emission tomography/computed tomography with 18F-FDG, depending on clinical indications. Genetic profiling of resected tumor tissue was performed by an external commercial laboratory. Demographic and clinical characteristics of the cohort are presented in Table 1; detailed depersonalized data for each study participant are provided in Table S1.

### 2.2. Collection and Preanalytical Processing of Biomaterials

Prior to surgical treatment (~60 min before surgery), patients donated 12 mL of peripheral venous blood collected into EDTA-containing vacuum tubes. Within 4 h after collection (storage at +4 °C), blood samples were centrifuged for 12 min at 3000× *g*. Then, plasma was separated and centrifuged again for 12 min at 12,000× *g* to remove any leftover microscopic cellular fragments. Purified plasma samples (5 mL) were immediately frozen at −80 °C.

At the beginning of surgery, prior to any potentially tissue-damaging procedures, 2.5–5.0 mL of CSF was collected using lumbar puncture. CSF samples were immediately frozen at −80 °C. All CSF samples were visually inspected for blood contamination, and no visible blood contamination was observed in any sample. RBC counts were not determined because the available CSF volume was prioritized for DNA extraction to maximize cfDNA recovery, given the anticipated low cfDNA concentrations in this biofluid. Consequently, no RBC count–based exclusion criterion was applied. After thawing, plasma and CSF samples were thoroughly mixed by pulse vortexing. cfDNA was extracted from 5 mL of plasma and 2.5–5.0 mL of CSF using the MagPure Circulating DNA Maxi Kit (Guangzhou Magen Biotechnology Co., Ltd., Guangzhou, China) on the Auto-Pure 24 instrument (Hangzhou Allsheng Instruments Co., Ltd., Hangzhou, China), according to the manufacturer’s instructions. The elution volume was set to 100 µL. Purified DNA samples were stored at −80 °C. Due to the addition of carrier RNA during cfDNA extraction, DNA quality was not assessed spectrophotometrically. Instead, the presence of double-stranded cfDNA in each sample was verified using the QuDye dsDNA HS Assay Kit (Lumiprobe GmbH, Hannover, Germany) on a Fluo-200 spectrofluorometer (Hangzhou Allsheng Instruments Co., Ltd., Hangzhou, China).

### 2.3. ddPCR: Conditions, Quality Control, and Data Interpretation

Droplet generation and fluorescence reading were performed using the QX200 AutoDG ddPCR System (Bio-Rad Laboratories, Inc., Hercules, CA, USA). ddPCR amplification was performed using a Veriti Thermal Cycler (Thermo Fisher Scientific, Inc., Waltham, MA, USA).

ddPCR reaction mixtures contained ddPCR Supermix for Probes (Bio-Rad Laboratories, Inc., Hercules, CA, USA), 9.9 µL of cfDNA, and 20× primers/probes mixtures. The sequences and concentrations of all oligonucleotides used in the mutation detection assays are provided in Table 2. Optimal annealing/extension temperatures were established in temperature-gradient experiments.

The thermocycling protocol for the *pTERT* C228T + C250T assay (analyzed in a single well) was as follows: enzyme activation at 95 °C for 10 min, followed by 55 cycles of denaturation at 94 °C for 30 s and annealing/extension at 54 °C for 2 min, and enzyme deactivation at 98 °C for 10 min. The thermocycling conditions for the *IDH1* R132H assay were slightly different: enzyme activation at 95 °C for 10 min, followed by 50 cycles of denaturation at 94 °C for 30 s and annealing/extension at 54 °C for 1 min, and enzyme deactivation at 98 °C for 10 min.

Each cfDNA sample was analyzed in a single technical replicate. A minimum of 10,000 accepted droplets per well was required for analysis. ddPCR was repeated for wells that did not meet this quality criterion. Inter-run variability was controlled by assessing variation in mutant allele fraction (MAF) in a mutation-positive DNA sample; acceptable variation was defined as <10%. For both assays each run included two mutation-positive control samples, two mutation-negative control samples, and two nucleic acid-free negative control samples.

The *pTERT* C228T + C250T assay was extensively validated previously using mutation-positive DNA samples and synthetic DNA constructs carrying the target mutations [22,23,24]. The assay enables simultaneous detection of *pTERT* C228T and C250T mutations using a single mutant probe; therefore, discrimination between these two mutations was not possible. The assay is characterized by a limit of blank of 0.01% and a limit of detection of 0.1% MAF.

The *IDH1* R132H assay was designed using OligoArchitect web-based software tool (v4.0, Sigma-Aldrich Co., Ltd., St. Louis, MO, USA). The assay was validated using serial dilutions of wild-type DNA and mutation-positive DNA samples. The results were concordant with the ddPCR™ Mutation Assay: *IDH1* p.R132H (Bio-Rad Laboratories, Inc., Hercules, CA, USA) (*p* > 0.05). The assay is characterized by a limit of blank of 0.02% and a limit of detection of 0.1% MAF.

It is worth noting that the reported analytical limits of detection were 0.1% MAF under the evaluated validation conditions. These values were not independently established using cfDNA-like fragmented templates at the molecular inputs encountered in the present study.

ddPCR results were analyzed using QuantaSoft 1.7 software (Bio-Rad Laboratories, Inc., Hercules, CA, USA). Droplet cluster separation in two-dimensional plots was performed manually by a blinded operator based on positive and negative control samples included in each batch. Results were reported as cftDNA concentration (copies of cfDNA carrying at least one of the assayed mutations per milliliter of biomaterial) and MAF (%). MAF was calculated using the following equation:

(1) MAF = C_mutated DNA_/(C_wild type DNA_ + C_mutated DNA_) × 100%,

where “C” is the DNA concentration in copies/μL of the ddPCR mixture. Total cfDNA concentration was estimated using the *IDH1* R132H ddPCR assay by summing the concentrations of wild-type and R132H-mutant *IDH1* target copies. Target concentrations were normalized to the original plasma or CSF volume, accounting for the total ddPCR reaction volume, the volume of DNA eluate added to the reaction, the total elution volume, and the biofluid volume used for DNA extraction. The combined wild-type and mutant copy concentration was divided by two, assuming two *IDH1* copies per diploid genome, and expressed as diploid genome equivalents per milliliter. This parameter therefore estimates total amplifiable cfDNA, irrespective of whether the *IDH1* R132H mutation was detected. dMIQE2020 checklist for presenting quantitative ddPCR experiments is available in Table S2.

### 2.4. Statistical Analysis

IBM SPSS Statistics 26.0 software (IBM Corp., Armonk, NY, USA) was used for statistical analysis. Data distribution was assessed using the Shapiro–Wilk test. Because none of the variables demonstrated a normal distribution, nonparametric statistical methods were applied. Paired continuous variables were compared using the Wilcoxon signed-rank test, whereas unpaired continuous variables were compared using the Mann–Whitney U test. Paired categorical variables were compared using McNemar’s test, whereas unpaired categorical variables were compared using Fisher’s exact test or Fisher–Freeman–Halton exact test for more than two groups. Correlations between variables were assessed using Spearman’s rank correlation coefficient (ρ).

Due to the low detection rate of ctDNA observed in this study, continuous variables are presented as median [5th percentile; 95th percentile]. A *p*-value < 0.05 was considered statistically significant. The Benjamini–Hochberg procedure was applied separately within three hypothesis families: overall plasma–CSF comparisons; mutation-specific comparisons among tumor tissue, CSF, and plasma; and exploratory association and correlation analyses involving cfDNA, clinical, and demographic variables. The false discovery rate was set to 0.10 within each family. *p*-values below 0.05 that did not pass the threshold for this correction for multiple comparisons were marked with an asterisk “*”.

## 3. Results

### 3.1. Comparison of cfDNA Characteristics Between Plasma and CSF

cfDNA samples were available for analysis from 65 CSF specimens and 63 plasma specimens, of which 62 specimens were paired. Results of cfDNA analysis in each sample are presented in Table S1, whereas examples of representative 2D ddPCR plots are available in Figure S1.

**Figure 1:**
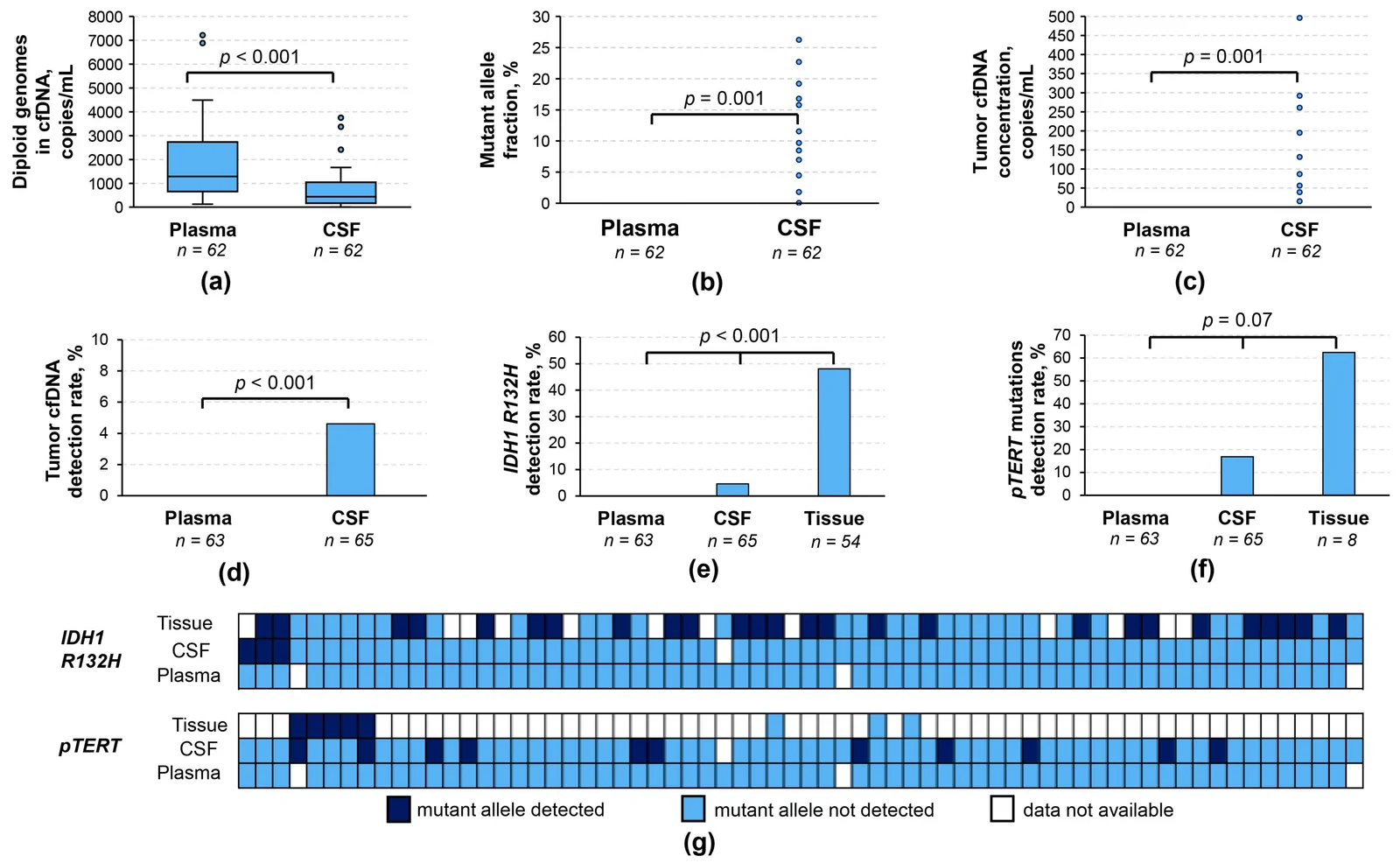
Comparison of cfDNA levels for paired plasma and CSF samples and overall detection rates in unpaired plasma, CSF, and tissue samples. (**a**) diploid genomes in cfDNA, copies/mL; (**b**) MAFs, %; (**c**) tumor cfDNA concentrations, copies/mL; (**d**) tumor cfDNA detection rate, %; (**e**) *IDH1* R132H mutation detection rate, %; (**f**) *TERT* promoter (C228T and C250T) mutations detection rate, %; (**g**) binary heatmap showing the presence of *IDH1* R132H and *TERT* promoter mutations in tissue, CSF, and plasma. Pairwise comparisons of detection rates are available in Table S3. Horizontal axis presents individual samples. Tumor cfDNA is defined as DNA harboring *IDH1* R132H and/or *TERT* promoter (C228T and C250T) mutations. Data in (**a**–**c**) are shown as box plots: center line—median; box limits—25th and 75th percentiles; whiskers—minimum to maximum (excluding outliers). Outliers are shown as individual points. Extreme outliers (>8000 copies/mL for plasma and >4000 copies/mL for CSF) were excluded to preserve proportions of the figure. All *p*-values below 0.05 successfully passed the correction for multiple comparisons (Benjamini–Hochberg procedure at false discovery rate of 0.10). Abbreviations: cfDNA, cell-free DNA; CSF, cerebrospinal fluid; MAF, mutant allele fraction.

The concentration of total cfDNA, expressed as diploid genome equivalents, was significantly lower in CSF than in plasma: 434.1 [19.4; 12,440.5] versus 1240.3 [193.5; 24,074.5] copies/mL, respectively (*p* < 0.001) (Figure 1a). In contrast, cftDNA carrying at least one of the analyzed mutations was detected significantly more frequently in CSF than in plasma. The cftDNA detection rate was 21.5% (14/65) in CSF, whereas no cftDNA-positive plasma samples were identified (0/63; 0.0%) (*p* < 0.001) (Figure 1d).

Consequently, the distribution of cftDNA concentrations was also significantly higher in CSF than in plasma: 0.0 [0.0; 466.7] vs. 0.0 [0.0; 0.0] copies/mL, respectively (*p* = 0.001) (Figure 1c). The same was true for the differences in MAF values between paired CSF and plasma samples: 0.0 [0.0; 18.8] vs. 0.0 [0.0; 0.0]%, respectively (*p* = 0.001) (Figure 1b). Although the median cftDNA concentration and MAF were zero in both biofluids because most samples were mutation-negative, their overall distributions differed significantly.

### 3.2. Detection of Individual Mutations in Plasma, CSF, and Tumor Tissue

Among patients with paired tumor tissue and CSF results, *IDH1* R132H and *pTERT* mutations were detected in CSF in 2/26 (7.7%) and 2/5 (40.0%) tissue-positive cases, respectively (Table 3). No mutations were detected in plasma among tissue-positive patients (*IDH1* R132H, 0/26; *pTERT*, 0/4), and the *pTERT* estimates should be interpreted cautiously because of the small number of evaluable pairs. Pairwise comparisons of detection rates for both hotspots in plasma, CSF, and tumor tissue are available in Table S3.

The overall detection rate of the *IDH1* R132H mutation differed significantly among plasma, CSF, and tumor tissue samples (Figure 1e). This mutation was not detected in plasma (0/63; 0.0%), whereas it was identified in 3 of 65 CSF samples (4.6%) and 26 of 54 tumor tissue samples (48.1%; *p* < 0.001).

The overall combined detection rate of the *pTERT* C228T and C250T mutations also differed among the three sample types, although the difference did not reach the significance threshold (Figure 1f). At least one of these mutations was detected in 0 of 63 plasma samples (0.0%), 11 of 65 CSF samples (16.9%), and 5 of 8 tumor tissue samples with available *pTERT* mutation results (62.5%; *p* = 0.07).

Among patients for whom paired CSF and tumor tissue results were available, every mutation detected in CSF was also identified in the corresponding tumor tissue. No mutation was detected in CSF in the presence of a mutation-negative paired tumor tissue sample. A sample-by-sample visualization of mutated DNA detection rates for each type of biomaterial is presented in Figure 1g.

### 3.3. Association of cfDNA Characteristics with Clinical and Demographic Parameters

**Figure 2:**
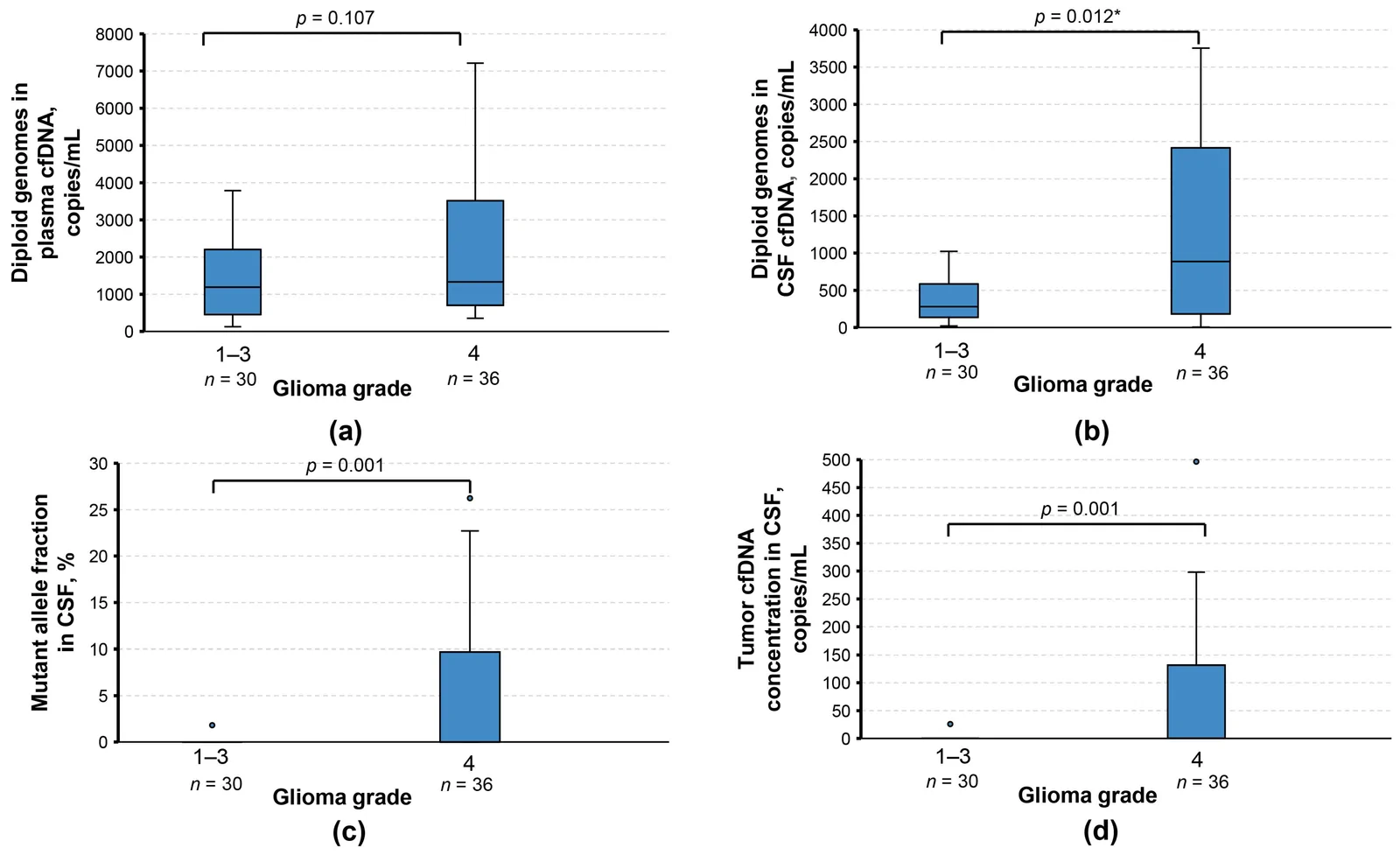
Comparison of quantitative cfDNA parameters in plasma and CSF between glioma patients with different tumor grades. (**a**) diploid genomes in plasma cfDNA, copies/mL; (**b**) diploid genomes in CSF cfDNA, copies/mL; (**c**) MAFs in CSF, %; (**d**) tumor cfDNA concentrations in CSF, copies/mL. Tumor cfDNA is defined as DNA harboring *IDH1 R132H* and/or *TERT* promoter (C228T and C250T) mutations. Data are shown as box plots: center line—median; box limits—25th and 75th percentiles; whiskers—minimum to maximum (excluding outliers). Outliers are shown as individual points. Extreme outliers (>8000 copies/mL for plasma and >4000 copies/mL for CSF) were excluded to preserve proportions of the figure. * This *p*-value did not pass the correction for multiple comparisons (Benjamini–Hochberg procedure at false discovery rate of 0.10). Abbreviations: cfDNA, cell-free DNA; CSF, cerebrospinal fluid; MAF, mutant allele fraction.

The concentration of diploid genome equivalents in plasma did not differ significantly between patients with grade 4 gliomas and those with grade 1–3 gliomas: 1312.5 [439.6; 107,319.0] vs. 1192.5 [145.6; 23,253.3] copies/mL, respectively (*p* = 0.107) (Figure 2a). In contrast, analysis of cfDNA in CSF revealed that patients with grade 4 gliomas had higher concentrations of diploid genome equivalents: 888.8 [8.0; 16,425.8] vs. 270.0 [38.3; 968.1] copies/mL, respectively; however, its *p*-value did not pass the correction for multiple comparisons (*p* = 0.012*) (Figure 2b). Similarly, cftDNA concentrations and MAFs were higher in grade 4 cases than in the rest: 0.0 [0.0; 1209.3] vs. 0.0 [0.0; 12.9] copies/mL and 0.0 [0.0; 23.8] vs. 0.0 [0.0; 0.9] %, respectively (*p* = 0.001) (Figure 2c,d).

**Figure 3:**
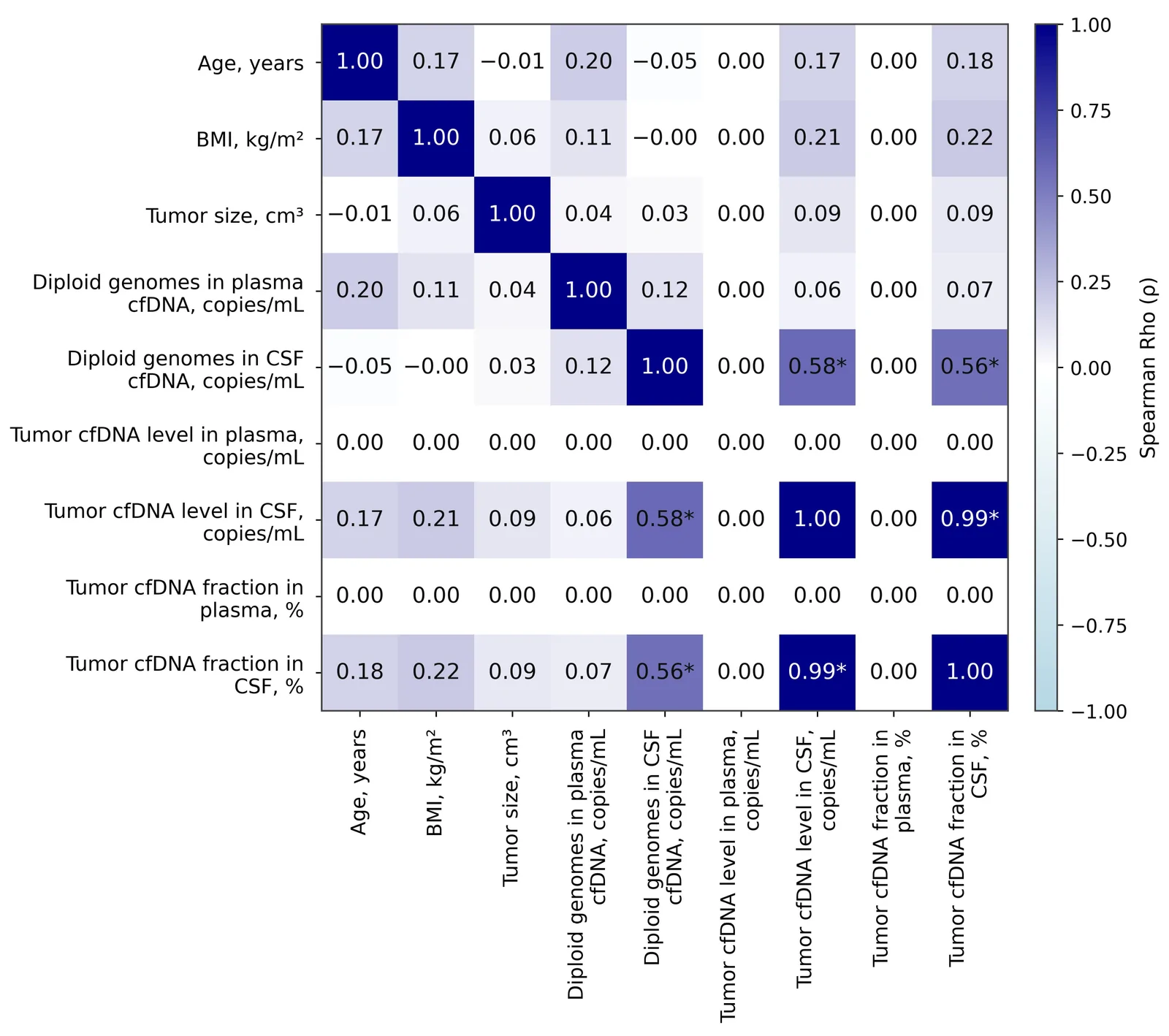
Spearman correlation matrix of clinical and cfDNA parameters in glioma patients. Positive correlations are shown in blue, negative correlations in light blue. Statistically significant correlations (*p* < 0.05), which passed successfully passed the correction for multiple comparisons (Benjamini–Hochberg procedure at false discovery rate of 0.10) are marked with an asterisk (*). Abbreviations: cfDNA, cell-free DNA; CSF, cerebrospinal fluid; BMI, body mass index.

CSF diploid genome-equivalent concentration, cftDNA concentration, and MAF were not significantly correlated with tumor size, plasma diploid genome-equivalent concentration, patient age, or body mass index (all *p* > 0.05). However, the concentration of diploid genome equivalents in CSF demonstrated a moderate positive correlation with CSF MAF and cftDNA concentration (Spearman’s ρ = 0.58, *p* < 0.001). Results of Spearman’s correlation analysis for all included variables are presented in Figure 3.

**Figure 4:**
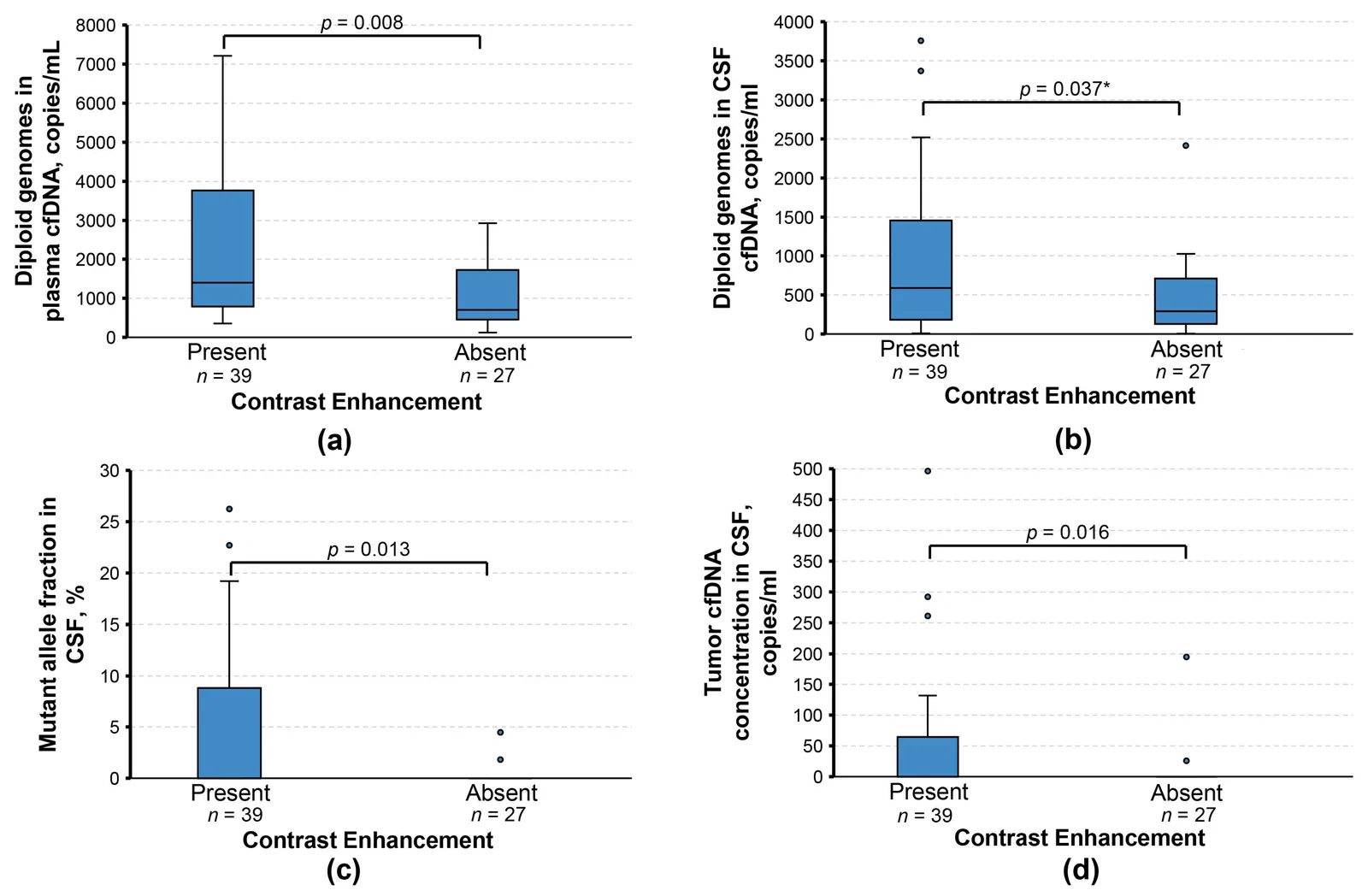
Comparison of quantitative cfDNA parameters in plasma and CSF between glioma patients with and without contrast enhancement. (**a**) diploid genomes in plasma cfDNA, copies/mL; (**b**) diploid genomes in CSF cfDNA, copies/mL; (**c**) MAFs in CSF, %; (**d**) tumor cfDNA concentrations in CSF, copies/mL. Tumor cfDNA is defined as DNA harboring *IDH1 R132H* and/or *TERT* promoter (C228T and C250T) mutations. Data are shown as box plots: center line—median; box limits—25th and 75th percentiles; whiskers—minimum to maximum (excluding outliers). Outliers are shown as individual points. Extreme outliers (>8000 copies/mL for plasma and >4000 copies/mL for CSF) were excluded to preserve proportions of the figures. * This *p*-value did not pass the correction for multiple comparisons (Benjamini–Hochberg procedure at false discovery rate of 0.10). Abbreviations: cfDNA, cell-free DNA; MAF, mutant allele fraction, CSF, cerebrospinal fluid.

Patients whose tumors demonstrated contrast enhancement had significantly higher concentrations of diploid genome equivalents in plasma than patients whose tumors did not demonstrate it: 1378.1 [406.1; 86,072.6] vs. 704.1 [139.4; 16,902.3] copies/mL, respectively (*p* = 0.008) (Figure 4a). The concentration of diploid genome equivalents in CSF was also higher in patients with contrast-enhancing tumors: 590.1 [17.4; 16,016.8] vs. 281.0 [11.6; 1909.3] copies/mL in patients without contrast enhancement, although the *p*-value did not pass the correction for multiple comparisons (*p* = 0.037*) (Figure 4b). The same observation was made for cftDNA concentrations and MAFs in CSF: 0.0 [0.0; 974.4] versus 0.0 [0.0; 135.8] copies/mL and 0.0 [0.0; 23.2] versus 0.0 [0.0; 0.9]%, respectively (*p* = 0.016 and 0.013) (Figure 4c,d).

Finally, tumor localization was not significantly associated with diploid genome-equivalent concentration, cftDNA concentration, or MAF in either plasma or CSF (all *p* > 0.05).

## 4. Discussion

In this study, the *IDH1* R132H mutation and the *pTERT* C228T and C250T mutations were selected as analytical targets because they are among the most recurrent and diagnostically informative hotspot alterations in adult gliomas and collectively represent several major molecular subgroups of these tumors. *IDH1* or *IDH2* mutations occur in more than 70% of adult diffuse gliomas, with *IDH1* R132H accounting for the large majority of *IDH*-mutant cases [5]. *TERT* promoter mutations show a complementary distribution: contemporary WHO-defined series have reported them in approximately 90–95% of oligodendrogliomas, about 66% of *IDH*-wildtype glioblastomas, and fewer than 10–20% of IDH-mutant astrocytomas [6,25]. In our cohort, *IDH1* R132H was detected in 48.1% (26/54) of patients with available tumor specimens. This frequency appears compatible with the mixed composition of the cohort, which included gliomas of grades 1–4 and in which grade 4 tumors constituted more than half of all cases. Such a cohort was expected to contain both *IDH*-mutant astrocytomas and a substantial proportion of *IDH*-wildtype glioblastomas [3]. The *pTERT* C228T/C250T tissue detection rate of 62.5% was likewise within the broad range expected for an adult cohort enriched in grade 4 disease. However, this estimate was based on only eight tissue-tested patients and therefore should not be interpreted as a reliable estimate of *pTERT* mutation prevalence in the entire cohort. Thus, the tissue findings broadly support the biological relevance of the selected targets, but the restricted tissue denominator, particularly for *pTERT*, precludes detailed comparison of mutation frequencies among histomolecular entities.

The most striking finding was the complete absence of detectable cftDNA carrying *IDH1* R132H or *pTERT* hotspot mutations in all 63 analyzed plasma specimens. Our result is consistent with the very low plasma yield reported by Fontanilles et al., who detected circulating *pTERT* mutations in only 2 of 52 patients with newly diagnosed glioblastoma, both of whom had gliosarcoma [20]. A similarly low cftDNA detection rate was reported by Juratli et al., who detected *pTERT* mutations in only 3 of 38 plasma samples from patients with *pTERT* mutation-positive tumor tissue [7]. In contrast, Muralidharan et al. reported a *pTERT* mutation-detection sensitivity of 62.5% and specificity of 90%, and Jones et al. detected *IDH1* R132H with 77% sensitivity in preoperative samples and reported detection rates of 88% and 49% for *pTERT* C228T and C250T, respectively (the overall specificity was approximately 90%) [21,26]. Crucitta et al. more recently demonstrated a plasma *IDH1* R132H sensitivity of 47.6% with specificity of 89.1%, although their blood samples were collected after surgery [27]. Differences in tumor selection, timing of blood collection, plasma volume, extraction recovery, amplicon design, preamplification, number of replicate reactions, positivity thresholds, and whether sensitivity was calculated only among tissue-positive patients can plausibly account for much of this interstudy variability.

The blood–brain barrier probably contributed to these results, although it should not be regarded as a uniform or completely impermeable boundary in glioma. Gliomas contain a spatially heterogeneous blood–tumor barrier: permeability is commonly increased within contrast-enhancing regions, whereas infiltrative non-enhancing margins may retain relatively intact barrier properties [28]. Tumor-derived DNA that does reach the systemic circulation is additionally diluted by abundant cfDNA released from hematopoietic and other non-neoplastic tissues and is subject to rapid clearance [13]. Several methods were developed to increase the permeability of the barrier, namely transient hyperosmotic disruption with intra-arterial mannitol and localized opening by focused ultrasound in combination with intravenously administered microbubbles [29,30]. Importantly for liquid biopsy research, first in human studies have shown that focused-ultrasound-mediated barrier opening can transiently enrich circulating brain-derived biomarkers, and the related “sonobiopsy” concept has now been evaluated prospectively in patients with high-grade glioma [31,32]. These approaches remain experimental and would not currently be justified solely to obtain a diagnostic blood sample, but they demonstrate that anatomical compartmentalization is a modifiable determinant of plasma biomarker yield.

Methodological factors in our study probably also reduced mutation detection yield in plasma. The 105-bp *IDH1* amplicon was suitable for fragmented cfDNA, whereas the 160-bp *pTERT* amplicon was close to the length of a mononucleosomal plasma fragment and necessarily excluded target molecules in which the entire sequence between the two primers was not preserved [13]. The longer amplicon was required because of the GC-rich *pTERT* sequence. The 160-bp *pTERT* amplicon may have limited detection in fragmented cfDNA because amplification requires preservation of the complete target region, including both primer-binding sites. Fragment-size distributions were not measured in this cohort, and no dedicated validation or recovery experiments using cfDNA-like fragmented templates were performed. In CSF, wild-type target concentrations measured with the *pTERT* assay were significantly lower than those measured with the *IDH1* R132H assay (medians: 296 vs. 882 copies/mL; 65 paired samples; *p* < 0.001). In plasma, the corresponding medians were 1956.4 and 2576.2 copies/mL, respectively, but the paired difference did not reach statistical significance (63 paired samples; *p* = 0.053). These findings provide analytical context but do not establish fragmentation as their cause or validate sensitivity for rare mutant molecules. The lower median wild-type target concentrations measured with the *pTERT* assay compared with the *IDH1* assay are compatible with reduced availability of amplifiable *pTERT* templates, but this comparison cannot distinguish fragmentation effects from other assay-specific differences or establish sensitivity for rare mutant molecules. Accordingly, the previously reported *pTERT* limit of detection of 0.1% MAF should be interpreted within the original validation conditions. Its applicability to fragmented specimens at the molecular inputs encountered here remains unestablished. The absence of detectable plasma mutations may therefore reflect both biological and analytical constraints, and the observed detection rates describe the performance of the present workflow.

Furthermore, cfDNA extracted from 5 mL of plasma was eluted in 100 µL, of which 9.9 µL was analyzed in a single ddPCR well. At the observed median concentration of 1240.3 diploid genome equivalents/mL, a simple template-counting estimate corresponds to approximately 600 genome equivalents entering one reaction, assuming an even distribution throughout the eluate. At a true MAF of 0.1%, fewer than one mutant template would be expected on average.

In contrast to plasma, cftDNA was detected in 21.5% of CSF specimens, even though the median concentration of total diploid genome equivalents in CSF was approximately threefold lower than in plasma. CSF contains less background DNA overall but may be selectively enriched for DNA released within the central nervous system. It is worth noting that in this study CSF was sampled preoperatively, before any tissue damaging procedures, which enabled an unbiased comparison of plasma and CSF. The moderate positive correlation between total CSF diploid genome-equivalent concentration and MAF (Spearman’s ρ = 0.58) is consistent with the proposition that at least part of the increase in total CSF cfDNA was tumor-derived, although the correlation alone cannot exclude contributions from non-neoplastic neural, inflammatory, or blood-derived cells.

Several biological mechanisms may favor CSF detection. cftDNA can enter adjacent interstitial fluid and CSF without first traversing the blood–brain barrier; the probability of release is expected to rise with apoptosis, necrosis, cellular turnover, pial or ependymal invasion, and direct contact with ventricular or subarachnoid spaces. CSF also contains substantially less leukocyte-derived wild-type DNA than plasma, improving the relative representation of tumor molecules [33]. Fragmentation remains an important analytical constraint. Glioma-associated CSF cfDNA is highly fragmented and is enriched for fragments shorter than 150 bp [34]. Consequently, the 105-bp *IDH1* assay should have been compatible with a larger fraction of CSF molecules than the 160-bp *pTERT* assay. The lower analytical limit of detection of the *pTERT* assay and the higher prevalence of *pTERT* mutations in the relevant tumors may nevertheless have partly compensated for this size disadvantage. Low absolute template input probably remained a major limiting factor. At the median CSF concentration of 434.1 diploid genome equivalents/mL, extraction from 2.5–5.0 mL and analysis of 9.9 µL of the 100-µL eluate would place only approximately 100–200 genome equivalents into a typical reaction. At a 1% MAF, this corresponds to approximately one or two mutant molecules.

Our overall CSF detection rate was lower than the 74% reported by Wang et al., the 85% reported in the sequencing study by Martínez-Ricarte et al., the approximately 49% reported by Miller et al., and the 71% diagnostic rate reported with chip-based digital PCR by Fujioka et al. [18,19,35,36]. These studies were not directly comparable with ours: several used tumor-informed variants or multigene sequencing panels, included patients with progressive or advanced disease, analyzed ventricular or cisternal CSF, or selected tumors adjacent to a CSF reservoir. Indeed, Wang et al. detected tumor DNA in all evaluable high-grade tumors abutting a CSF space but in none of the tumors without such contact [18]. In contrast, our fixed three-mutation panel was applied to an unselected mixed-grade surgical cohort, irrespective of whether the tumor carried one of the targets or contacted a CSF compartment. The 21.5% positivity rate should therefore be interpreted as the yield of this specific targeted workflow rather than as the true biological prevalence of cftDNA in glioma CSF.

Some of the differences between our findings and those generated in NGS-based studies are likely attributable to the analytical approach. In this study, ddPCR was the analytical method of choice due to it being particularly well suited for the analysis of predefined hotspot variants in samples containing small amounts of highly fragmented DNA. Compared with conventional quantitative PCR, ddPCR generally offers greater precision at low target concentrations and more robust quantification of low-frequency targets [37,38]. Whereas compared with next-generation sequencing (NGS), targeted ddPCR has a shorter workflow, requires less complex bioinformatic analysis, and can be more cost-effective when only a limited number of predefined mutations are investigated, although it does not permit the discovery of unexpected genomic alterations [39,40]. Conversely, broad NGS panels can aggregate evidence across multiple somatic variants and thereby achieve a higher sample-level detection rate, although this advantage depends on adequate library complexity, sequencing depth, molecular barcoding, and error suppression. For the primary objective of our study, ddPCR remained an appropriate method because paired plasma and CSF samples were processed and interrogated with the same assays, reaction volumes, quality criteria, and positivity framework. The marked within-cohort difference between the two biofluids is therefore less susceptible to platform-related confounding than comparisons of absolute detection rates across studies.

Preamplification has been proposed as an effective strategy for improving the detection of low-abundance targets in ddPCR, including cftDNA in plasma and CSF [41,42,43]. In our previous study, preamplification of CSF cfDNA increased the sensitivity of *IDH1* R132H mutation detection by 36%; however, this improvement was accompanied by an 18% decrease in specificity, likely related to the so-called splashome effect during batch processing and analysis of preamplified samples [15]. In the present study, we therefore chose not to include a preamplification step. Although this decision may have reduced the probability of detecting very low-abundance mutant molecules, we considered minimizing false-positive results to be particularly important in the context of molecular profiling of adult gliomas and thus prioritized analytical specificity over a potential gain in sensitivity.

Tumor grade was one of the clinical characteristics most clearly associated with CSF cfDNA findings. Patients with grade 4 gliomas had higher total diploid genome-equivalent concentrations, cftDNA concentrations, and MAFs in CSF than patients with grade 1–3 tumors, whereas total plasma cfDNA did not differ significantly between these groups. This pattern is concordant with studies in which higher glioma grade, greater disease burden, radiological progression, or dissemination along ventricular and subarachnoid surfaces increased the probability of detecting CSF tumor DNA [18,35,36]. Several features of grade 4 gliomas could contribute mechanistically. These tumors typically show high cellularity and proliferative activity, increased apoptosis and necrosis, microvascular proliferation, abnormal vascular permeability, and invasive growth, all of which can increase DNA release or facilitate access to extracellular fluid compartments [3,44]. Molecular subtype is an important potential confounder because the prevalence of the analytical targets is not uniform across grades: *pTERT* mutations are frequent in *IDH*-wildtype glioblastoma, whereas *IDH1* R132H is concentrated in *IDH*-mutant astrocytomas and oligodendrogliomas [3]. Thus, the grade-related difference may reflect both more efficient DNA release from aggressive tumors and differences in the probability that a tumor carried one of the selected mutations. The comparison also combined biologically heterogeneous grade 1, 2, and 3 tumors into one group, and the limited number of cftDNA-positive cases did not permit robust multivariable adjustment for confounders. Notably, Jones et al. reported an association between total plasma cfDNA and glioma grade, whereas our cohort showed the grade-related signal only in CSF [21]. This discrepancy further suggests that plasma cfDNA is influenced by cohort composition and systemic as well as tumor-specific sources, while CSF may more directly reflect local central nervous system tumor biology.

Contrast enhancement was likewise associated with higher total cfDNA concentrations in both plasma and CSF and with higher cftDNA concentrations and MAFs in CSF. Gadolinium enhancement reflects extravasation of contrast through a disrupted blood–tumor barrier and is commonly associated with abnormal angiogenesis, enlarged and permeable vessels, inflammation, hypoxia, and necrosis [45]. These characteristics provide plausible routes by which an enhancing tumor could release more DNA. In CSF, contrast enhancement is unlikely to be the direct route by which DNA enters the fluid, but it can act as an imaging marker of the same aggressive, necrotic, and highly vascular phenotype that promotes local DNA shedding. Several studies similarly identified contrast enhancement as a predictor of successful mutation detection in CSF [7,36,46]. Our mutation-specific CSF findings are therefore consistent with the literature. By contrast, the higher total plasma cfDNA concentration in enhancing tumors should be interpreted cautiously because no target mutation was detected in plasma; the excess plasma DNA could have arisen partly or entirely from non-neoplastic tissues or systemic responses to advanced disease. Because the present sample size and number of positive CSF samples did not support a fully adjusted model, our results do not establish contrast enhancement as an independent determinant of cftDNA release.

Neither tumor size nor anatomical localization was significantly associated with the cfDNA parameters measured in plasma or CSF. Published evidence regarding these relationships is heterogeneous. Some studies have associated cftDNA detection with overall tumor burden or progression, whereas others indicate that direct contact with a CSF reservoir, ventricular or subarachnoid dissemination, and quantitative barrier permeability are more important than conventional tumor diameter or volume [18,35,47]. The absence of an association in our cohort therefore does not necessarily conflict with prior observations. Future analyses would benefit from quantitative MRI variables such as enhancing and necrotic volumes, permeability metrics, tumor-to-CSF distance, and explicit ventricular, ependymal, or subarachnoid contact. The present null findings should be interpreted as an absence of demonstrable associations under the applied classification scheme rather than evidence that tumor anatomy and burden are biologically irrelevant.

This study has several strengths, including paired preoperative sampling, collection of CSF before tissue-damaging surgical procedures, identical targeted analysis of both biofluids, analytically validated assays, and the absence of discordant CSF-positive/tissue-negative findings among evaluable pairs. Nevertheless, several limitations should be acknowledged. To begin, this was a single-center study with a modest cohort of 66 patients; consequently, subgroup estimates were imprecise and multivariable modeling of potential confounders was restricted. Next, the cohort combined several glioma grades and molecular entities, while comprehensive WHO-integrated molecular classification was unavailable for some participants. Furthermore, the panel covered only *IDH1* R132H and two *pTERT* hotspots. Thus, a negative result indicates only the absence of detectable selected targets and not the absence of cftDNA. Besides this, tissue profiling results were not available for all study participants, preventing reliable calculation of clinical sensitivity and specificity. The absence of a non-glioma control group also prevented direct estimation of clinical specificity in the intended diagnostic setting, although concordance with available tissue results provides some reassurance. Finally, the cross-sectional preoperative design did not assess postoperative kinetics, treatment response, recurrence, or prognostic value, and the imaging variables did not directly quantify tumor–CSF contact or barrier permeability.

## 5. Conclusions

In this study, paired preoperative plasma and lumbar CSF samples from patients with glioma were analyzed by ddPCR for the *IDH1* R132H and *pTERT* C228T and C250T mutations. Although the total concentration of cfDNA, expressed as diploid genome equivalents, was approximately threefold lower in CSF than in plasma, cftDNA carrying at least one of the selected mutations was detected in 21.5% of CSF samples and in none of the plasma samples. Furthermore, cftDNA concentrations and mutant allele fractions were significantly higher in CSF. Among patients with available paired molecular data, every mutation detected in CSF was also identified in the corresponding tumor tissue, supporting the specificity of the applied targeted assays.

The quantitative characteristics of CSF cfDNA were also associated with clinically relevant tumor features. Patients with grade 4 gliomas had higher total cfDNA concentrations, cftDNA concentrations, and mutant allele fractions in CSF than patients with grade 1–3 tumors. Similar associations were observed in patients with contrast-enhancing tumors. At the same time, tumor size and anatomical localization were not significantly associated with the evaluated cfDNA parameters. These findings suggest that cftDNA release into CSF may be influenced more strongly by biological aggressiveness, cellular turnover, necrosis, vascular abnormalities, and disruption of tissue barriers than by tumor size or lobar localization alone.

Overall, within the limitations of a single-center cohort, incomplete tissue molecular profiling, and analysis of only three predefined hotspot mutations, the principal finding of this study was that lumbar CSF was substantially more informative than plasma for detecting glioma-derived DNA using the present ddPCR workflow. The absence of detectable target mutations in plasma should not be interpreted as evidence that glioma-derived DNA is entirely absent from the circulation, because low template input and the restricted mutation panel may have limited cftDNA detection yield. CSF-based ddPCR may therefore represent a useful complementary approach for targeted molecular characterization of gliomas, particularly when suitable tumor tissue is unavailable, although it cannot currently replace comprehensive tissue profiling. Larger prospective multicenter studies incorporating complete paired tissue genotyping, broader multiplexed panels, shorter amplicons, increased molecular input, replicate reactions, standardized CSF processing, and longitudinal sampling are required to define the diagnostic and monitoring applications of CSF cftDNA analysis.

## Figures and Tables

**Table 1:** Demographic and clinical characteristics of study participants.

Parameters	Glioma Cohort (*n* = 66)
Age, years	49.17 (22.0–71.0)
Sex, *n* (%)	
- Male	28 (42.42%)
- Female	38 (57.58%)
BMI, kg/m^2^	25.96 (17.04–41.47)
Tumor size, cm^3^	48.3 [19.7; 88.0]
Tumor grade, *n* (%)	
- 1	2 (3.03%)
- 2	10 (15.15%)
- 3	18 (27.27%)
- 4	36 (54.55%)
Tumor size rank, *n* (%):	
- <50 cm^3^	35 (53.03%)
- 50–100 cm^3^	17 (25.75%)
- 100–150 cm^3^	9 (13.64%)
- >150 cm^3^	5 (7.58%)
Tumor localization, *n* (%)	
- Frontal	36 (54.55%)
- Temporal	31 (46.97%)
- Parietal	22 (33.33%)
- Occipital	9 (13.64%)
- Insular	19 (28.79%)
- Nuclei	2 (3.03%)
Contrast enhancement, *n* (%)	
- None (0)	27 (40.9%)
- Moderate (1)	15 (22.73%)
- Intense (2)	5 (7.58%)
- Ring-like (3)	19 (28.79%)
Mutations detected in tumor tissue, *n* (%)	
- *IDH1* R132H mutation *	26 (48.15%)
- *TERT* promoter mutations C228T and C250T *	5 (62.5%)
- 1p/19q codeletion *	12 (24.49%)
- *MGMT* promoter methylation *	20 (39.22%)

Continuous data are presented as mean (range) for age and BMI, and as median [quartile 1; quartile 3] for tumor size. * Tissue molecular testing was not available for all patients. Percentages are calculated from the number of patients with available data: *IDH1* R132H—26/54, *TERT* promoter—5/8, 1p/19q—12/49, *MGMT*—20/51. Abbreviations: BMI, body mass index; *IDH1*, isocitrate dehydrogenase 1; *MGMT*, O^6^-methylguanine-DNA methyltransferase; *TERT*, telomerase reverse transcriptase.

**Table 2:** Sequences of oligonucleotide primers and probes used for *TERT* promoter and *IDH1 R132H* mutation detection assays.

Gene/Mutation	Amplicon Length	Oligonucleotide	Sequence	Concentration *
*TERT* promoter C228T and C250T **	160 bp	Forward primer	AGC GCT GCC TGA AAC TCG	0.9 μM
		Reverse primer	CCT GCC CCT TCA CCT TCC AG	0.9 μM
		Mutation probe	FAM-CCC +C+T+T +CCG G-BHQ1	0.25 μM
		Wild-type probe	HEX-CCC C+C+T +CCG G-BHQ1	0.25 μM
*IDH1* R132H	105 bp	Forward primer	CCA ACA TGA CTT ACT TGA	0.9 μM
		Reverse primer	CAG AGA AGC CAT TAT CTG	0.9 μM
		Mutation probe	FAM-CCA TAA GCA TGA TGA CCT ATG ATG A-BHQ1	0.25 μM
		Wild-type probe	HEX-CCA TAA GCA TGA CGA CCT ATG ATG A-BHQ1	0.25 μM

LNA nucleotides are denoted with a “+” sign (+N). * Concentration in the final ddPCR mixture. ** TERT promoter mutations assay functions only with ddPCR supermixes containing dUTP. Abbreviations: FAM, 6-carboxyfluorescein; HEX, hexachlorofluorescein; BHQ1, Black Hole Quencher 1; bp, base pairs.

**Table 3:** Mutation-specific performance of CSF and plasma testing using tumor tissue mutation status as the reference standard.

**Mutation**	**Biofluid**	**Paired *n***	**TP**	**FN**	**TN**	**FP**	**Sensitivity, % (95% CI)**		**Specificity, % (95% CI)**
IDH1 R132H	CSF	53	2	24	27	0	7.7 (0.9–25.1)		100.0 (87.2–100.0)
IDH1 R132H	Plasma	51	0	26	25	0	0.0 (0.0–13.2)		100.0 (86.3–100.0)
TERT promoter	CSF	8	2	3	3	0	40.0 (5.3–85.3)		100.0 (29.2–100.0)
TERT promoter	Plasma	7	0	4	3	0	0.0 (0.0–60.2)		100.0 (29.2–100.0)
**Mutation**	**Biofluid**	**PPV, % (95% CI)**			**NPV, % (95% CI)**			**Overall Agreement, *n*/*N* (%)**	
IDH1 R132H	CSF	100.0 (15.8–100.0)			52.9 (38.5–67.1)			29/53 (54.7)	
IDH1 R132H	Plasma	Not estimable			49.0 (34.8–63.4)			25/51 (49.0)	
TERT promoter	CSF	100.0 (15.8–100.0)			50.0 (11.8–88.2)			5/8 (62.5)	
TERT promoter	Plasma	Not estimable			42.9 (9.9–81.6)			3/7 (42.9)	

Each comparison includes only patients with positive or negative results for the corresponding mutation in both tumor tissue and the specified biofluid. Missing specimens were excluded separately for each comparison. Confidence intervals are two-sided exact 95% Clopper–Pearson intervals. PPV was not estimable for plasma because there were no positive plasma results. Predictive values apply to the evaluable paired subsets. TERT promoter mutations (C228T and C250T) estimates are exploratory because of the limited paired sample sizes. Abbreviations: TP, tissue-positive/biofluid-positive; FN, tissue-positive/biofluid-negative; TN, tissue-negative/biofluid-negative; FP, tissue-negative/biofluid-positive; CI, confidence interval; PPV, positive predictive value; NPV, negative predictive value.

## Data Availability

All data generated in the present study are available in Table S1.

## Supplementary Materials

The following supporting information can be downloaded at https://www.mdpi.com/article/10.3390/diagnostics16203333/s1: Figure S1: Representative examples of 2D ddPCR plots for the reported mutation detection assays; Table S1: Depersonalized clinical, demographic, and experimental data for each study participant; Table S2: dMIQE2020 checklist for presenting quantitative ddPCR experiments; Table S3: Pairwise comparisons of *IDH1* R132H and *TERT* promoter mutation detection rates in tumor tissue, CSF, and plasma.

## Abbreviations

The following abbreviations are used in this manuscript:

cfDNA	Cell-free DNA
cftDNA	Cell-free tumor DNA
MAF	Mutant allele fraction
*pTERT*	Promoter of the *TERT* gene
PCR	Polymerase chain reaction
ddPCR	Digital droplet polymerase chain reaction
CSF	Cerebrospinal fluid
NGS	Next-generation sequencing
