# Supplementary material for: Comparative Detection of IDH1 R132H and TERT Promoter Mutations in Plasma and Cerebrospinal Fluid of Patients with Glioma Using Targeted Droplet Digital PCR
Source: Diagnostics (Basel). 2026 Oct 14;16(20):3333. doi: 10.3390/diagnostics16203333 (PMC13703826; doi:10.3390/diagnostics16203333)
Supplement: Supplementary file 1 [file diagnostics-16-03333-s001.zip › Figure S1. Representative examples of 2D ddPCR plots for the reported mutation detection assays.pdf]

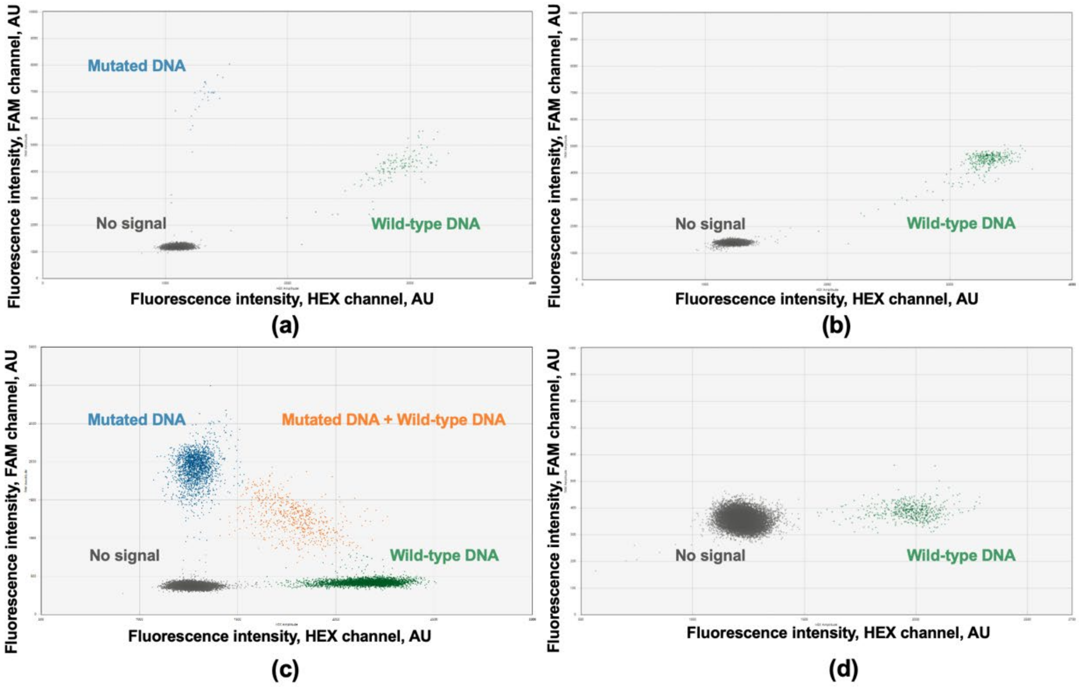

Figure S1. Representative examples of 2D ddPCR plots for the reported mutation detection assays: (a) positive for the *IDH1 R132H* mutation; (b) negative for the *IDH1 R132H* mutation; (c) positive for the *pTERT* mutations; (d) negative signal for the *pTERT* mutations. These examples were obtained from CSF cfDNA samples
