# Supplementary material for: Comparative Detection of IDH1 R132H and TERT Promoter Mutations in Plasma and Cerebrospinal Fluid of Patients with Glioma Using Targeted Droplet Digital PCR
Source: Diagnostics (Basel). 2026 Oct 14;16(20):3333. doi: 10.3390/diagnostics16203333 (PMC13703826; doi:10.3390/diagnostics16203333)
Supplement: Supplementary file 1 [file diagnostics-16-03333-s001.zip › Table S3_pairwise comparisons.pdf]

Table S3. Pairwise comparisons of *IDH1* R132H and *TERT* promoter mutation detection rates in tumor tissue, CSF, and plasma.

Mutation	Specimen comparison	Evaluable pairs, N	Positive in first specimen, n/N (%)	Positive in second specimen, n/N (%)	*p*-value
IDH1 R132H	Tumor tissue vs. CSF	53	26/53 (49.1%)	2/53 (3.8%)	< 0.001
IDH1 R132H	Tumor tissue vs. plasma	51	26/51 (51.0%)	0/51 (0.0%)	< 0.001
IDH1 R132H	CSF vs. plasma	62	3/62 (4.8%)	0/62 (0.0%)	0.250
TERT promoter	Tumor tissue vs. CSF	8	5/8 (62.5%)	2/8 (25.0%)	0.250
TERT promoter	Tumor tissue vs. plasma	7	4/7 (57.1%)	0/7 (0.0%)	0.125
TERT promoter	CSF vs. plasma	62	10/62 (16.1%)	0/62 (0.0%)	0.002

Each comparison includes only patients with available results for the corresponding mutation in both specified specimens. The first and second specimens follow the order shown in the comparison column. Values are presented as the number of mutation-positive results divided by the number of evaluable pairs, with percentages in parentheses. Missing or unperformed tests were excluded separately for each comparison and were not classified as negative. Detection rates were compared using the two-sided exact McNemar test. All *p*-values below 0.05 successfully passed the correction for multiple comparisons (Benja-mini-Hochberg procedure at false discovery rate of 0.10). Comparisons involving tumor tissue *TERT* promoter status (mutations C228T and C250T) should be interpreted cautiously because of the small numbers of evaluable pairs. CSF, cerebrospinal fluid.
